# Instability in the COPD Diagnosis upon Repeat Testing Vary with the Definition of COPD

**DOI:** 10.1371/journal.pone.0121832

**Published:** 2015-03-26

**Authors:** Rogelio Perez-Padilla, Fernando C. Wehrmeister, Maria Montes de Oca, Maria Victorina Lopez, Jose R. Jardim, Adriana Muino, Gonzalo Valdivia, Julio Pertuze, Ana Maria B. Menezes, PLATINO group

**Affiliations:** 1 Sleep Clinic, National Institute of Respiratory Diseases, Mexico City, Mexico; 2 Faculty of Medicine, University of the Republic, Montevideo, Uruguay; 3 Pulmonary Division, Hospital Universitario de Caracas, Universidad Central de Venezuela, Caracas, Venezuela; 4 Postgraduate program on Epidemiology, Federal University of Pelotas, Pelotas, Brazil; 5 Pontificia Universidad Catolica de Chile, Santiago, Chile; 6 Federal University of Sao Paulo, Sao Paulo, Brazil

## Abstract

**Background:**

A low FEV_1_/FVC from post-bronchodilator spirometry is required to diagnose COPD. Both the FEV_1_ and the FVC can vary over time; therefore, individuals can be given a diagnosis of mild COPD at one visit, but have normal spirometry during the next appointment, even without an intervention.

**Methods:**

We analyzed two population-based surveys of adults with spirometry carried out for the same individuals 5-9 years after their baseline examination. We determined the factors associated with a change in the spirometry interpretation from one exam to the next utilizing different criteria commonly used to diagnose COPD.

**Results:**

The rate of an inconsistent diagnosis of mild COPD was 11.7% using FEV_1_/FVC <0.70, 5.9% using FEV_1_/FEV_6_ <the lower limit of the normal range, LLN and 4.1% using the GOLD stage 2-4 criterion. The most important factor associated with diagnostic inconsistency was the closeness of the ratio to the LLN during the first examination. Inconsistency decreased with a lower FEV_1_.

**Conclusions:**

Using FEV_1_/FEV_6_ <LLN or GOLD stage 2-4 as the criterion for airflow obstruction reduces inconsistencies in the diagnosis of mild COPD. Further improvement could be obtained by defining a borderline zone around the LLN (e.g. plus or minus 0.6 SD), or repeating the test in patients with borderline results.

## Introduction

Population-based prevalence of poorly reversible airflow obstruction (COPD) has been recently estimated for a variety of countries in Latin America [1] and other continents [2] with standardized methods including post-bronchodilator spirometry. In those and other surveys, it has been clear that the several criteria to define airflow obstruction would produce important differences in COPD prevalence, which would be lower with criteria based on FEV_1_/FVC or FEV_1_/FEV_6_ below the 5th percentile (lower limit of normal, LLN), than with the traditional Global Initiative for Obstructive Lung Disease (GOLD) definition (FEV_1_/FVC<0.7). The majority of the emphasis has been placed on cross-sectional exams and on the impact of COPD criteria on population prevalence, but consistency of diagnosis is also very important for these persons because false positives label them as having a potentially severe disease and false negatives could lead to denial of useful treatments. Additional complications would derive from inconsistency of diagnosis after serial spirometric tests due to known and expected variations in spirometric results after repeated testing that may change the diagnosis along time[3] adding to systematic changes due to aging, and to worsening or improvements in airflow obstruction. In a population-based survey, COPD prevalence based on FEV_1_/FEV_6_ <LLN showed more consistency than criteria based on FEV_1_/FVC as the former has fixed times to determine the numerator and denominator of the ratio, whereas FEV_1_/FVC only fixes the time to measure the numerator and variations in the expiratory time change the airflow obstruction prevalence [4].

We hypothesized that there would be instability in the designation of abnormal spirometric results depending on the COPD definition used. Therefore, we conducted this study to determine the frequency of change in the diagnosis in the same subjects studied in two population-based surveys, as well as the correlates for these inconsistencies.

## Methods

The detailed methods of the PLATINO baseline[5] and follow-up studies [6] are available elsewhere. Between the years 2003 and 2005, population-based surveys were conducted employing standardized methodology in five large Latin-American metropolitan areas: Sao Paulo (Brazil), Mexico City (Mexico), Montevideo (Uruguay), Santiago (Chile), and Caracas (Venezuela). We successfully interviewed 1,000 subjects aged 40 years or older in Sao Paulo, 1,063 in Mexico City, 943 in Montevideo, 1,208 in Santiago, and 1,357 in Caracas. Spirometry testing was performed for 963 (97.9%) subjects in Sao Paulo, 1,000 (98.3%) in Mexico City, 885 (97.1%) in Montevideo, 1,173 (99.8%) in Santiago, and 1,294 (98.4%) in Caracas[1]. The questionnaire is available at the PLATINO website: http://www.platino-alat.org. Ethical approval was obtained from the Institutional Review Boards at all five sites (Instituto Nacional de Enfermedades Respiratorias, Universidad Central de Venezuela, Pontificia Universidad Católica de Chile, Federal University of Pelotas and Federal University of Sao Paulo) for the baseline exam and at the three sites participating in the follow-up exams. All participants signed a written informed consent approved by the Boards.

Spirometry was undertaken in individuals who did not present any exclusion criteria (99% of the sample) using an ultrasonic spirometer (EasyOne; ndd Medical Technologies, Zurich, Switzerland). Spirometry was performed before (pre-BD) and 15 minutes after the administration of 200 μg of Salbutamol (post-BD) according to the American Thoracic Society (ATS) criteria of acceptability and reproducibility [7]. The quality control exercises showed that >90% of the tests fulfilled ATS quality criteria [5].

Follow-up studies were conducted in Montevideo, Santiago, and Sao Paulo 5, 6, and 9 years after the baseline surveys, respectively[6]. Only individuals with valid spirometric data at baseline were eligible for a follow-up exam. Individuals were visited at their homes based on the contact information provided by these during the baseline exam. For the purposes of the present study, only individuals with valid postbronchodilator spirometric tests during both surveys were analyzed.

Data analyses contained the description of the sample characteristics and the calculation of the intraclass correlation coefficient (ICC Rho) between the spirometric parameters (as a continuous variable) in the two tests and the concordance in the diagnosis of COPD (as dichotomic data using the ICC Rho and the Kappa coefficient) with the following criteria: a) LLN [8–10]—defined as the lower 5^th^ percentile for predicted post-BD FEV_1_/FVC based on equations derived from the baseline study in a sub-set of healthy and never smoking subjects [11]; b) a ratio of the post-BD FEV_1_ over FVC < 0.70 according to the Global Initiative for Obstructive Lung Disease (GOLD)[12,13]; c) FEV_1_/FVC_6_ <LLN defined as the lower 5^th^ percentile for predicted post-BD FEV_1_/FEV_6_ based on PLATINO reference equations [11]; d) GOLD stages 2–4 defined as FEV_1_/FVC<0.7 & FEV_1_<80% predicted, that has been used increasingly to add specificity, and similar indices based on LLN, e.g. FEV_1_/FVC & FEV_1_<LLN, and FEV_1_/FEV_6_ & FEV_1_<LLN.

The probability of an inconsistent spirometric diagnosis during the second examination (one with COPD and the other with no COPD) versus consistent results (both with the same diagnosis) was estimated from logistic regression models, including as independent variables the deviation of the measured FEV_1_/FVC and FEV_1_/FEV_6_ (in standard deviations [SD] from predicted) from the LLN and, in addition, adjustment by several confounders (forced expiratory time, age, gender, current smoking, cumulative smoking in pack-years, the presence of respiratory symptoms, and previous physician diagnosis of respiratory diseases obtained from a questionnaire). Weight and height were measured, and Body mass index (BMI) was categorized into two groups: normal and overweight/obesity. Although in the present work we do not analyze adverse outcomes in detail[14], we investigated if indices giving less inconsistencies on repeated testing also better predicted risk of death and the risk of two or more exacerbations in the previous year. In the presence of airflow obstruction (for the different definitions) we estimated risk of death during follow-up from proportional hazard models (Cox regression done in all individuals including those who died and did not performed the second spirometry) and risk of more than 2 exacerbations in the previous year with a logistic regression model taking into account age and gender. All of the analyses were adjusted for study site (country) and were stratified by gender.

## Results

Table 1 describes the main baseline characteristics of the study participants. Follow-up exams were conducted for 885 adults in Montevideo, 1,173 in Santiago, and 963 in Sao Paulo. Information was obtained for 758 (85.6%), 993 (84.7%) and 748 (77.7%) subjects, respectively, of whom 2,026 had a good quality post-BD spirometry test during both examinations (68.8% of those with post bronchodilator testing in the first evaluation and 75.6% of those with post bronchodilator testing in the first evaluation surviving or lost at the time of the second evaluation) (see Table 2). Follow-up rates for each independent variable category were around 80% [5,6]

**Table 1:** Characteristics of the 2,026 adults who participated in both examinations and had a valid spirometry test.

	First exam		Second exam	
	Mean (%)	SD (or 95%CI)	Mean (%)	SD (or 95%CI)
Men (95%CI)	40.6	39.0–42.2	-	-
Age (years)	55.6	10.9	62.2	10.8
Height (cm)	160.4	9.7	159.8	9.8
BMI (Kg/m^2^)	28.3	5.7	28.8	5.3
FEV_1_ pre-BD (L)	2.65	0.78	2.42	0.77
FVC pre-BD (L)	3.56	0.99	3.24	0.97
FEV_1_ post-BD (L)	2.74	0.77	2.50	0.77
FVC post-BD (L)	3.54	0.96	3.26	0.96
FEV_1_/FVC pre-BD	74.3	8.2	74.7	8.5
FEV_1_/FVC post-BD	77.7	8.1	76.8	8.5
FEV_1_/FEV_6_ pre-BD	78.1	6.2	77.7	6.9
FEV_1_/FEV_6_ post-BD	80.4	6.2	79.2	6.8
COV FEV_1_/FVC pre-BD	0.80	0.37–1.47	0.78	0.34–1.44
COV FEV_1_/FVC post-BD	0.67	0.30–1.25	0.68	0.31–1.34
COV FEV_1_/FEV_6_ pre-BD	0.47	0.23–0.91	0.45	0.20–0.86
COV FEV_1_/FEV_6_ post-BD	0.42	0.19–0.76	0.45	0.20–0.86
Good quality pre-BD* (95%CI)	90	89–92	83	81–85
Good quality post-BD* (95%CI)	91	90–93	79	77–81
FET pre-BD (s)	11.7	3.9	9.9	3.9
FET post-BD (s)	10.3	3.4	9.4	3.7
History of Asthma (95%CI)	16.4	14.6–18.1	16.0	14.1–17.1
History of COPD (95%CI)	4.2	3.3–5.1	6.2	5.1–7.3
Current smoker (95%CI)	31.1	28.8–33.5	25.3	23.2–27.4

BMI = weight/ height^2^; COV = Coefficient of variability, expressed as medians, 25th and 75th percentiles. SD = Standard deviation; FET = forced expiratory time; pre-BD = before bronchodilator; post-BD = after Bronchodilator. Good quality = three acceptable tests with two best FEV_1_ and FVC within <150mL. LLN = lower limit of normal; 95%CI = 95% confidence interval.

**Table 2:** Population eligible, spirometries done, individuals switching COPD categories and percentage with 2 or more exacerbations according to several definitions of airflow obstruction (COPD).

Criteria	Number with post BD spirometry at baseline	Dead at follow up	Alive or lost at follow up	Number with post BD spirometry at follow up	Number switching COPD categories (%)	Percentage with 2+ exacerbations in previous year at 2nd evaluation (95%CI)
FEV_1_/FVC<0.7 (GOLD)	502	96 (19.1)	406 (80.9)	300 (73.9)	91 (30.3)	2.4 (1.3–4.2)
Remaining	2440	169 (6.9)	2271 (93.1)	1726 (76.0)	146 (8.5)	2.1 (1.6–2.8)
FEV_1_/FVC <LLN	274	50 (18.2)	224 (81.8)	167 (74.6)	68 (40.7)	2.9 (1.5–5.7)
Remaining	2668	215 (8.1)	2453 (91.9)	1859 (75.8)	63 (3.4)	2.1 (1.6–2.7)
FEV_1_/FEV_6_ <LLN	251	48 (19.1)	203 (80.9)	149 (73.4)	45 (30.2)	3.6 (1.9–6.8)
Remaining	2691	217 (8.1)	2474(91.9)	1877 (75.9)	86 (4.6)	2.0 (1.6–2.7)
GOLD 2–4	191	47 (24.6)	144 (75.4)	110 (76.4)	29 (26.4)	4.2 (2.1–8.2)
Remaining	2751	218 (7.9)	2533 (92.1)	1916 (75.6)	54 (2.8)	2.0 (1.6–2.6)
FEV_1_/FVC & FEV_1_<LLN	101	25 (24.8)	76 (75.2)	58 (76.3)	21 (36)	7.9 (4.0–15.1)
Remaining	2841	240 (8.4)	2601 (91.6)	1968 (69.3)	23 (1.2)	2.0 (1.5–2.6)
FEV_1_/FEV_6_ & FEV_1_<LLN	106	25 (23.6)	81 (76.4)	61 (75.3)	20 (32.8)	7.5 (3.8–14.4)
Remaining	2836	240 (8.5)	2596 (91.5)	1965 (69.3)	34 (1.7)	2.0 (1.5–2.6)

PostBD = post bronchodilator; Switched categories includes the number and the percentage with reference to previous column spirometry at follow up. 2026 subjects with two spirometries; GOLD 2–4 = FEV_1_/FVC<0.7 & FEV_1_<80% predicted; LLN = lower limit of normal, the 5th percentile adjusted for gender, age and height. 95%CI = 95% confidence intervaL

Compared with the first examination, individuals with a follow-up exam had less current smoking, and with slightly lower lung function. Mean Forced expiratory time (FET) decreased by about 1 second but was >6 seconds on average, and the rate of valid tests decreased from 90% to 83%. The coefficient of variability of FEV_1_/FEV_6_ was lower than that of FEV_1_/FVC, both before and after BD in both examinations.

**Fig 1:**
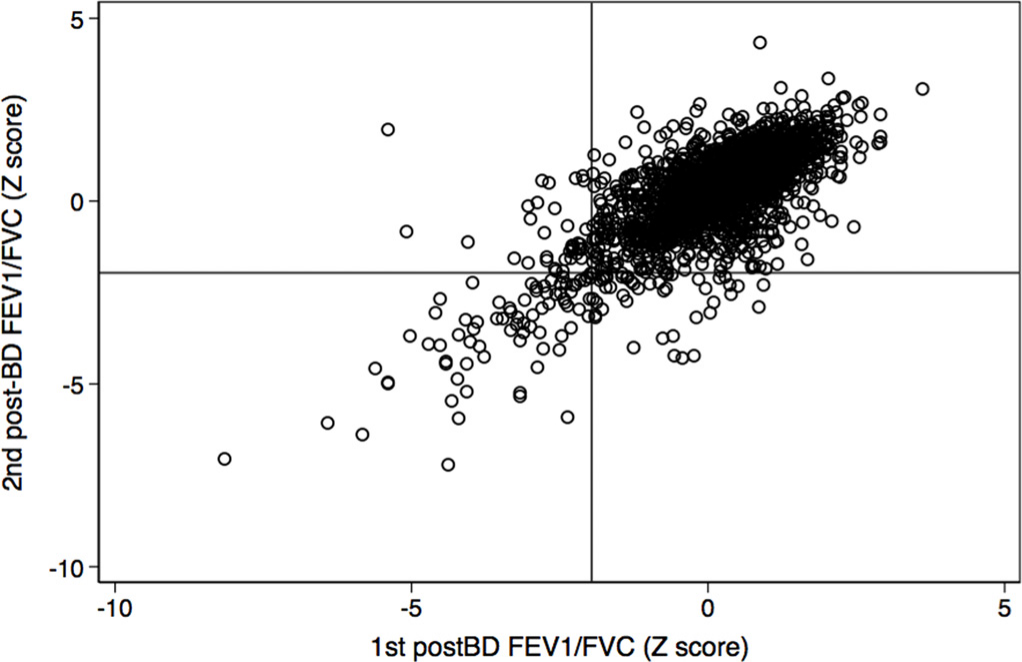
Post-Bronchodilator (BD) FEV_1_/FVC in the first vs. the second examination, both expressed as Z- score of the PLATINO predicted values. Lines at -1.645 represent the 5th percentile of the measurement (LLN). Compare with Fig. 2, and observe that dispersion is worse for FEV_1_/FVC (Rho concordance 0.74) than for FEV_1_/FEV_6_ (Rho concordance 0.82).

**Fig 2:**
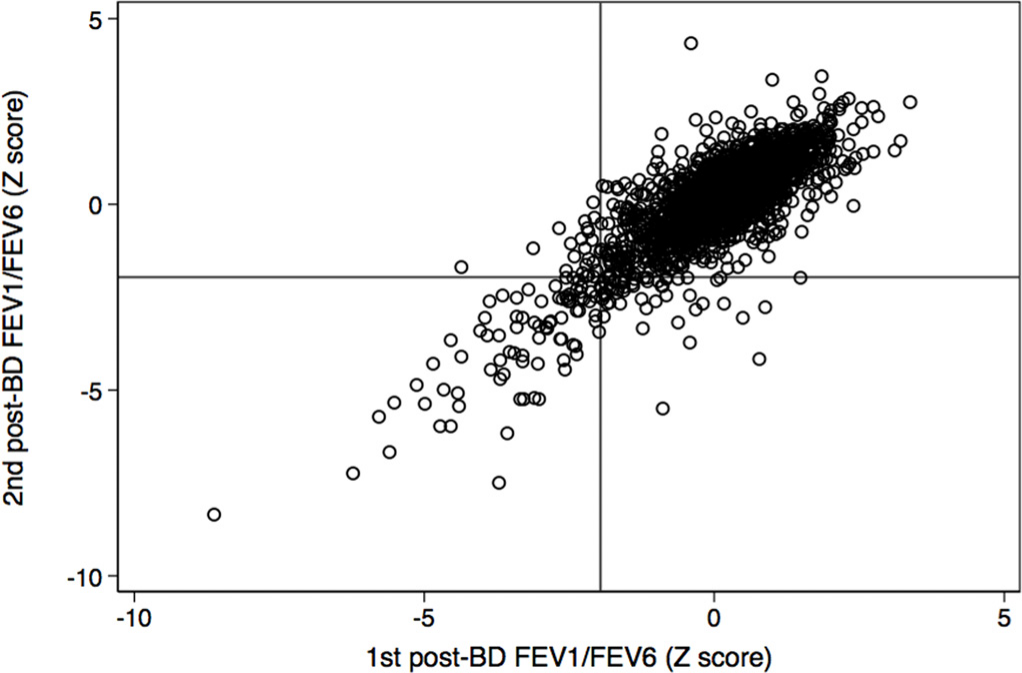
Post-bronchodilator (BD) FEV_1_/FEV_6_ in the first versus the second examination, both expressed as Z- score of the PLATINO predicted values. Lines at -1.645 represent the 5th percentile of the measurement (LLN). Compare with Fig. 1, and observe that dispersion is worse for FEV_1_/FVC (Rho concordance 0.74) than for FEV_1_/FEV_6_ (Rho concordance 0.82).

Fig. 1 depicts a scatter plot of the FEV_1_/FVC and Fig. 2 the FEV_1_/FEV_6_ both expressed as Z-scores of the PLATINO predicted values.

Dispersion is narrower with FEV_1_/FEV_6_ (Intraclass correlation coefficient Rho 0.81, 95%CI 0.78–0.84) when compared to the FEV_1_/FVC (ICC Rho 0.77, 95%CI 0.74–0.79). Reliability for for diagnosis (FEV_1_/FVC or FEV_1_/FEV_6_ as a dichotomic variable) measured similarly by the ICC Rho were much lower numerically than the ICC Rho coefficients for original variables: for GOLD criteria was 0.57 (95%CI 0.54–0.60), for GOLD stages 2–4 was 0.64 (95%CI 0.61.0.66), for FEV_1_/FEV_6_<LLN was 0.64 (95%CI 0.61–0.66) and for FEV_1_/FVC<LLN 0.57 (95%CI 0.61–0.66) (S1 Table).

Inconsistent diagnoses occurred with all three criteria, but were more common when utilizing the GOLD criterion. Tables 2 and 3 describes the individuals switching spirometric diagnoses in the two examinations according to the airflow obstruction criteria as a proportion those with and without airflow obstruction (Table 2) or as a proportion of the total population (Table 3). Population inconsistencies were highest for GOLD criterion (11.7%), intermediate for FEV_1_/FVC<LLN (6.5%), and lowest for the FEV_1_/FEV_6_ <LLN criterion (5.9%). Prevalence of airflow obstruction and inconsistency in diagnosis was further reduced using indices requiring a low FEV_1_ in addition to a low ratio: GOLD-2–4 (4.1%), FEV_1_/FVC &FEV_1_<LLN (2.2%) AND FEV_1_/FEV_6_ & FEV_1_<LLN (2.7%) (See Table 3). In Table 3 also note that in general indicators giving the lowest inconsistency in longitudinal diagnosis also provided the lowest population prevalence, the highest hazard ratio for mortality in Cox regression models and the highest risk of having 2 or more exacerbations in the previous year adjusting by age and gender. In addition, in logistic regression models (adjusted by age and gender), individuals with airflow obstruction in both evaluations had increased risk of exacerbations compared to those with inconsistent airflow obstruction regardless of the criterion of airflow obstruction. For example those with FEV_1_/FVC<LLN in both evaluations had an OR 2.99 (95%CI 1.2–7.3) of having 2+ exacerbations in the previous year, compared to an OR of 1.6 (0.6–4.0) in those with inconsistent obstruction. Similarly individuals with FEV_1_/FEV_6_<LLN in both evaluations had an OR of 3.6 (1.6–7.9) of 2+ exacerbations, vs. 1.8 (0.7–4.5) in those obstructed only in one evaluation. However, the association disappeared if FEV_1_ expressed as percentage of predicted was included in the models. All individuals with FEV_1_<50% predicted were in the consistent COPD diagnosis.

If inconsistency were expressed as percentage of airflow obstruction prevalence at first evaluation (Table 2 and column 6/column 2 from Table 3), differences among indices would be minor. In addition although prevalence of airflow obstruction may be similar in both evaluations, "abnormal" individuals are not necessarily the same: some of them had a "new diagnosis" at the second evaluation and some "normalized" (see Table 2). For example, from the total of individuals with COPD diagnosis at first examination (see Table 2), 30% would be reverted at second evaluation if using GOLD criteria, 26% if using GOLD stages 2–4, and 33% if using FEV_1_/FEV_6_ & FEV_1_<LLN.

**Table 3:** Prevalences of airflow obstruction, frequency of inconsistent longitudinal diagnosis and hazard ratio depending on the definition.

Criteria	COPD 1	95%CI	COPD 2	95%CI	Inconsistency (%)	95%CI	Hazard ratio*	95%CI	EXACERBATIONS (OR)	95%CI
FEV_1_/FVC<0.7 (GOLD)	14.8	13.2–16.4	17.5	15.9–19.2	11.7	10.3–13.1	1.77	1.3–2.4	1.9	0.9–3.9
FEV_1_/FVC <LLN	8.2	7.0–9.4	8.0	6.8–9.2	6.5	5.4–7.5	1.53	1.1–2.2	1.9	0.8–3.9
FEV_1_/FEV_6_ <LLN	7.4	6.2–8.5	10.2	8.9–11.5	5.9	4.8–6.9	1.70	1.2–2.4	2.5	1.1–5.0
GOLD 2–4	5.4	4.4–6.4	6.6	5.5–7.7	4.1	3.2–5.0	2.49	1.8–3.5	3.1	1.4–7.2
FEV_1_/FVC & FEV_1_<LLN	2.8	2.0–3.5	3.5	2.7–4.3	2.2	1.5–2.8	3.06	1.9–4.9	5.4	2.4–13.1
FEV_1_/FEV_6_ & FEV_1_<LLN	3.0	2.2–3.7	4.1	3.2–4.9	2.7	2.0–3.4	2.92	1.8–4.6	5.2	2.2–12.1

COPD 1 and COPD 2 = sample prevalence (as percentage) of COPD in the first and second evaluation in the 2026 subjects with two spirometries; GOLD 2–4 = FEV_1_/FVC<0.7 & FEV_1_<80% predicted; LLN = lower limit of normal, the 5th percentile adjusted for gender, age and height. Hazard ratio = the risk of death (n = 2348) from a Cox model adjusting by age and gender based on the presence of airflow obstruction depending on the definition used. Inconsistency is the percentage of participants with a change in diagnosis of airflow obstruction (either a new diagnosis in the second evaluation or the presence of airflow obstruction in the first evaluation but not in the second). Exacerbations = odds ratio of having 2 or more exacerbations in the previous year assessed at the second evaluation in a model adjusted by age and gender. 95%CI = 95% confidence interval.

A new diagnosis of COPD was observed in 7.3% (GOLD), 3.2% (FEV_1_/FVC<LLN) and 4.4% (FEV_1_/FEV_6_ <LLN) of the studied population, whereas a reversed diagnosis of COPD was observed in 4.4% (GOLD), 3.3% (FEV_1_/FVC<LLN), and 1.8% (FEV_1_/FEV_6_ <LLN). Variations of both the numerator and the denominator (FEV_1_/FVC or FEV_1_/FEV_6_) could explain that of the ratios and therefore the diagnosis of airflow obstruction. The majority of individuals with new airflow diagnosis had a drop of FEV_1_ in the second evaluation (from 68–82% depending on the criteria) and a minority an increase in FVC or FEV_6_ (18 to 34%). In the individuals whose airflow obstruction normalized in the second evaluation this was due (70–94%) to a decrease in FVC or FEV_6_ (the denominator) whereas an increase in FEV_1_ was observed less frequently (17–48%).

A decrease in FEV_1_ was associated with an increase in BMI, continuous smoking, and use of bronchodilators or corticosteroids whereas an increase in FVC or FEV_6_ was associated with a decrease in BMI or lack of use or bronchodilators or corticosteroids but predicting variables explained less than 3% of the variation of the change in FVC, FEV_6_ or FEV_1_. Therefore >97% of changes in FEV_1_, FVC or FEV_6_ were unaccounted for by the collected variables. Incident airflow obstruction was associated consistently with age and continuous smoking regardless of the definition but elimination of airflow obstruction was also associated with increased age. Again the variability of the change in spirometric measurements explained by the models was <3%.

If a borderline “buffer zone” is constructed 0.6 SD above and below the LLN of FEV_1_/FEV_6_, this would include 68% of measurements during the first examination and 63% of the measurements during the second examination. None of the individuals would have changed from no COPD in the first examination to COPD in the second examination (performed 5–9 years later), and only four individuals would have changed from COPD in the first exam to no COPD during the second exam. Therefore, nearly all of the changes in diagnosis would fell within the borderline zone. In addition, in this large borderline zone, only 7/1383 individuals (0.5%) had an FEV_1_<80% predicted (GOLD stage 2) and none had an FEV_1_ <50% predicted (GOLD stages 3 or 4). The obstructed category was 16% of the total, and included all GOLD stage 3–4 individuals whereas the normal category was also 16% of the total. The borderline zone could be constructed of different sizes, balancing the proportion of false COPD positives and the proportion of individuals with borderline results.

**Fig 3:**
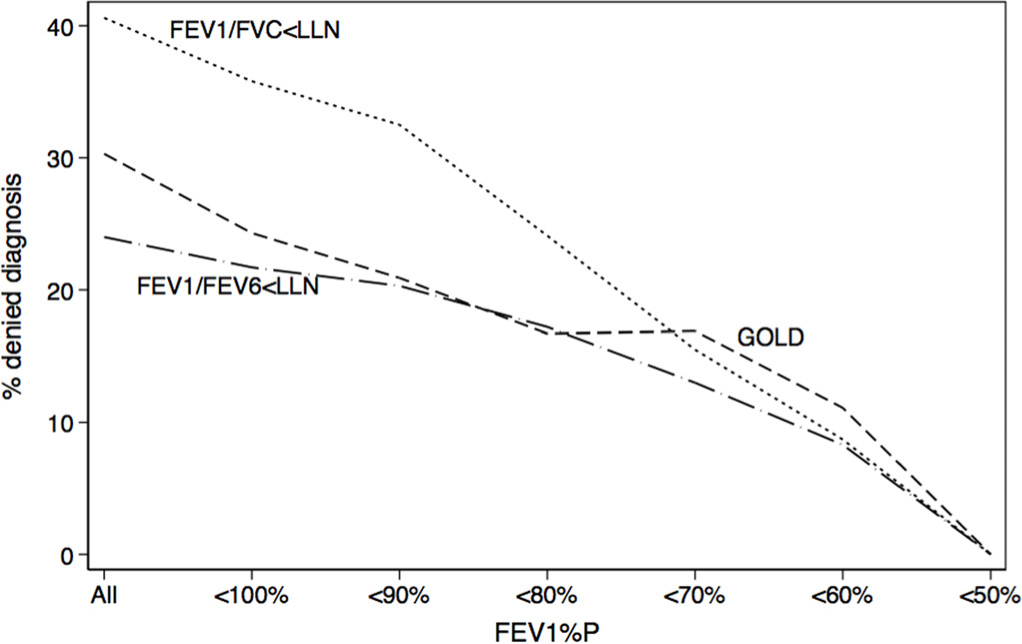
Cancelled diagnosis of airflow obstruction as percentage (vertical axis) decrease with lower FEV_1_ (horizontal axis) regardless of the criteria used. The graph depicts the results for three common criteria: GOLD (dashed line), FEV_1_/FVC<LLN (pointed line) and FEV_1_/FEV_6_<LLN (continuous line). The percentage of diagnostic inconsistencies in the population depend on the specificity of the criterion (see Tables).

In the logistic regression model (table 4), inconsistency was associated very powerfully with absolute distance of FEV_1_/FVC or FEV_1_/FEV_6_ (expressed as Z- scores) from the LLN. That is, the closer the measured FEV_1_/FVC or FEV_1_/FEV_6_ to the LLN, the higher the probability of an inconsistent diagnosis during the second examination. For example, the Odds ratio (OR) for an inconsistent diagnosis was 12 (95%CI 8–18) for FEV_1_/FVC<LLN, 28 (95%CI 18–45) for FEV_1_/FEV_6_ <LLN and 7 (95%CI 5–9) for GOLD criterion if the first result was <0.6 SD (above or below) from the LLN, compared to the remaining participants. Also, the lower the FEV_1_, the lower the inconsistency (OR 0.65–0.68 per SD from normality, see Table 5 and Fig. 3).

**Table 4:** Predicting inconsistency in airflow obstruction diagnosis by a logistic regression model.

	Inconsistency in FEV_1_/FVC<LLN			Inconsistency in FEV_1_/FEV_6_<LLN		
	Odds Ratio	95% CI	P	Odds Ratio	95% CI	P
Distance of FEV_1_/FVC or FEV_1_/FEV_6_ from the LLN (standard deviations)						
2–3	7.8	0.9–64	0.06	0.9	0.2–4.8	0.9
1 <2	9.6	1.2–76	0.03	2.8	0.6–13	0.17
0.6 <1	39.1	5–312	<0.0000	8.5	1.9–38	0.005
<0.6	108	14–842	<0.00001	75.8	18–319	<0.00001
FEV_1_ post BD as score Z	0.68	0.55–0.85	<0.00001	0.75	0.60–0.96	0.02
FET (s) first evaluation	1.2	1.1–1.3	0.001	1.06	1.0–1.13	0.04
FET (s) second evaluation	1.1	1.03–1.1	<0.0001	1.02	0.97–1.07	0.45
Pseudo R^2^	0.33			0.38		

LLN = lower limit of normal, 5th percentile of the ratio. FET = forced expiratory time; Model was also adjusted by age, gender, asthma, current smoking at second evaluation, a physician diagnosis of asthma. PostBD = post bronchodilator. The closer the ratio and the FEV_1_ to the diagnostic threshold, the higher the risk of inconsistency on repeated testing.

**Table 5:** Inconsistencies in the COPD diagnosis (post bronchodilator spirometry) and some of the predisposing factors. Same participants in two surveys 5–9 years apart.

Category	Percentage	95%CI
**FEV**_**1**_ **/FVC<0.70 (GOLD) INCONSISTENT**	11.7	10.3–13.1
New GOLD	7.3	6.0–8.5
Drop in FEV_1_	67.8	60–75
Continue smoking	27.4	20–35
Increase in FVC	53.4	45–62
Use of inhaled medications	9.6	5–14
Physician diagnosed asthma	8.0	5–11
Reversed GOLD	4.4	3.5–5.3
Increase in FEV_1_	25.3	16–34
Use of inhaled medications	7.8	2–13
Quit smoking	29.1	10–49
Drop in FVC	94.5	90–99
Increase in weight	74.7	66–84
Physician diagnosed asthma	5.6	4–9
**FEV**_**1**_ **/FEV**_**6**_ **<LLN INCONSISTENT**	5.9	4.8–6.9
New FEV_1_/FEV_6_ <LLN	4.4	3.4–5.2
Drop in FEV_1_	81.9	72–92
Continue smoking	40.0	27–53
Increase in FVC	34.4	22–47
Use of inhaled medications	13.1	4–21
Physician diagnosed asthma	7.1	5–10
Reversed FEV_1_/FEV_6_ <LLN	1.8	1.2–2.4
Increase in FEV_1_	19.4	6–33
Use of inhaled medications	16.6	4–29
Quit smoking	25.0	1–63
Drop in FVC	91.7	82–100
Increase in weight	66.6	50–82
Physician diagnosed asthma	3.5	2–6
**FEV**_**1**_ **/FVC<LLN INCONSISTENT**	6.5	5.4–7.5
New FEV_1_/FVC<LLN	3.2	2.2–4.0
Drop in FEV_1_	69.8	58–81
Continue smoking	32.3	20–44
Increase in FVC	63.4	51–76
Use of inhaled medications	11.1	3–19
Physician diagnosed asthma	4.4	2.7–7.2
Reversed FEV_1_/FVC<LLN	3.3	2.5–4.1
Increase in FEV_1_	17.6	8–27
Use of inhaled medications	13.2	5–21
Quit smoking	31	11–53
Drop in FVC	94.1	88–99
Increase in weight	72.1	61–83
Physician diagnosed asthma	5.6	4–9

Percentages on the left part of columns are from the total studied population, whereas those on the right are strata (overlapping) from the above categories. 95%CI = 95% confidence interval; Inconsistent = different result between the two examinations. New GOLD = fulfilling FEV_1_/FVC<0.70 only during the second exam (similar for the other airflow obstruction criteria). Reversed GOLD = met GOLD criterion during the first examination, but not during the second examination; LLN = lower limit of normal

A longer forced expiratory time was also associated with inconsistent diagnosis (OR 1.06–1.22), and a history of wheezing or asthma in some models, but usually with a borderline statistical significance. Other variables analyzed were age, continuous smoking, BMI, gender, clinical diagnosis of COPD, use of respiratory medications and the duration of the expiratory maneuvers, but all together they explained about 7–17% of the variability of inconsistent diagnosis, and the statistical significance disappeared (except for forced expiratory time) once we included in the models the proximity of the spirometry measurements to the threshold for airflow obstruction and FEV_1_. The closeness of FEV_1_/FVC or FEV_1_/FEV_6_ to the LLN was the main predictor of switching diagnosis in the two evaluations whether it was a new airflow obstruction in the second evaluation or an airflow obstruction found in the first evaluation disappearing in the second (see S2 Table with information for FEV_1_/FEV_6_).

## Discussion

We have described the rates and correlates of inconsistent interpretations of airway obstruction in adults during population-based follow-up examinations according to several criteria of airflow obstruction. There were three important findings. Firstly, the inconsistency of COPD diagnosis was observed with all criteria because these are based on sharp cut-off points (the LLN or a FEV_1_/FVC<0.7 for the GOLD criteria plus FEV_1_<80% of predicted for GOLD stage 2–4 or FEV_1_<LLN): that is, we arbitrarily dichotomize a continuous variable (FEV_1_/FVC, FEV_1_/FEV_6_ for COPD or FEV_1_ for GOLD stages). Utilizing the GOLD criterion, an individual with an FEV_1_/FVC of 0.69 would be diagnosed with COPD, and during a follow-up exam, if the ratio were 0.7, the subject would no longer be diagnosed as having COPD. Intraclass correlation coefficient, a measure of concordance was lower numerically for COPD diagnosis than for FEV_1_/FVC or FEV_1_/FEV_6_ as continuous variables. The same problem exists with strict cut-off values based on LLN. Although certain inconsistency is expected, the recommended diagnosis of COPD assumes a single spirometry and therefore does not consider longitudinal inconsistency.

Secondly, airflow obstruction criteria leading to lower population prevalence also produce less inconsistency on repeated testing: the FEV_1_/FVC had less concordance in the two tests than FEV_1_/FEV_6_ whereas the consistency of COPD diagnosis was highest for FEV_1_/FEV_6_ <LLN and for definitions of airflow obstruction requiring a low FEV_1_ (GOLD stage 2–4).

Thirdly, the closeness of the FEV_1_/FVC, FEV_1_/FEV_6_ and FEV_1_ to the thresholds or cut points, the higher the possibility of a change in diagnosis on repeated testing (Table 5) independently if the switch represents a new airflow obstruction or a "reversed" airflow obstruction (S2 Table).

Previous studies have shown that the within-test reproducibility of FEV_1_/FEV_6_ based on 6-second maneuvers was smaller when compared with the traditional FEV_1_/FVC [9]. We previously reported that utilizing FEV_1_/FEV_6_ <LLN provided even more consistent estimates of COPD prevalence in the same populations as criteria based on FEV_1_/FVC (GOLD or <LLN). Therefore, we expected that at the individual level, using the 6-second maneuvers would also provide a consistent diagnostic performance from our baseline examination to a follow-up examination; however, the inconsistency rate was not reduced to zero. This may be reduced by adding an interpretation category of "borderline" COPD in other words, by creating a buffer zone immediately around the LLN, as suggested previously by the National Lung Health Education Program (NLHEP) in the United States [15]. This zone would reduce or avoid the changes in diagnosis from extreme groups i.e. from COPD to normal, or from normal to COPD, instead forcing the majority of changes to occur from and to the borderline zone. This strategy would increase confidence in spirometric diagnosis and would be relatively easy to implement compared to a more sound approach of keeping as continuous FEV_1_/FVC, FEV_1_/FEV_6_ and FEV_1_ and estimating risks of disease or adverse outcomes or likelihood of response to treatments.

Clinicians usually possess information on individual patients that is not available to Epidemiologists and therefore can use this information to estimate the pre-test disease probability. They can then consider the magnitude of the abnormality from the spirometry test results in order to estimate the post-test probability of COPD. They can also order follow-up tests that will further increase or decrease the probability of COPD, such as a Diffusing capacity (DLCO) test, an exhaled nitric oxide test, induced sputum for eosinophil counts, or a lung Computed tomography scan (looking for emphysema). Clinicians can also perform an asthma therapy trial to determine whether the patient responds, rendering asthma much more likely than COPD. Clinical practice guidelines for hypertension recommend that prior to prescribing blood pressure medication, 3–6 repeated measurements of blood pressure over weeks to months be performed [16]. Perhaps a similar conservative approach should be taken with spirometry for COPD, especially if the results fall within the borderline zone. For these borderline cases, a conservative approach seems adequate because a false COPD label entails costs and potential adverse effects for the patient, including unwarranted bronchodilator prescription or further expensive tests. On the other hand, missing a case of mild COPD has little consequence, as all smokers should be advised to quit smoking regardless of spirometric results, and the majority of drug treatments are meant for moderate or severe COPD.

The main strengths of this study include the population-based sampling, high quality of the spirometry tests (15), the measurement of post-BD spirometry during both examinations and the relatively high rates of follow-up after 6–9 years. Limitations of our study include the following: A lack of annual spirometry testing, which could have determined whether or not study participants were “rapid decliners” consistent with COPD progression [15]. Shifts from airway obstruction to normal spirometry during the 5–9 year period could have occurred due to factors that we did not measure, such as respiratory infections or exposure to irritants during the first examinations (but not during the second examination), smoking cessation, or treatment for asthma, COPD, or heart failure; or reductions in exposures causing bronchospasm at the time of the first examination. However, an inconsistent airflow obstruction diagnosis, with any definition was associated significantly with the proximity to the limit of normality and the expiratory time of the tests and therefore likely mostly determined by intrinsic properties of the tests (reliability) rather than true pathobiological processes. Finally, in the current work we did not analyze in detail adverse outcomes, but thresholds giving less prevalence of airflow obstruction, also give less inconsistencies and are associated to a greater risk of death and of exacerbations.

In conclusion, current criteria for airflow obstruction, even those based on age- and gender- adjusted FEV_1_/FVC or FEV_1_/FEV_6_ <LLN, and those requiring in addition a low FEV_1_ produce inconsistencies over time that can cause confusing changes in the COPD diagnosis more likely if tests are around the threshold defining airflow obstruction. Diagnosing airflow obstruction using the 6-second spirometry (FEV_1_/FEV_6_ <LLN) especially if FEV_1_ is also low, reduces these inconsistencies compared with criteria based on FEV_1_/FVC<LLN or GOLD criterion. Perhaps the inclusion in the diagnostic scheme of a “gray or borderline” zone around the LLN would minimize changes from disease to health or vice-versa. Finally, clinicians should utilize information about the patient to weigh the consequences of a false-positive versus a false-negative disease label before making clinical decisions.

## Supporting Information

S1 Dataset Dataset in Excel (XLS) format. (XLS)

S1 Table Reliability by the intraclass correlation coefficient (ICC) and 95%CI between two spirometry tests (at baseline and follow-up) in the same individuals. (DOCX)

S2 Table Determinants of new and reversed airflow obstruction diagnosis by a logistic regression model using as criteria FEV_1_/FEV_6_<LLN. (DOCX)

## References

[1] Menezes AM, Perez-Padilla R, Jardim JR, Muino A, Lopez MV, Valdivia G, et al (2005) Chronic obstructive pulmonary disease in five Latin American cities (the PLATINO study): a prevalence study. Lancet 366: 1875–1881. 16310554 10.1016/S0140-6736(05)67632-5

[2] Buist AS, McBurnie MA, Vollmer WM, Gillespie S, Burney P, Mannino DM, et al (2007) International variation in the prevalence of COPD (the BOLD Study): a population-based prevalence study. Lancet 370: 741–750. 17765523 10.1016/S0140-6736(07)61377-4

[3] Reddel HK, Roberts B, Crockett A, Thoonen B, Lucas A, Thamrin C, et al Lack of consistency over time of airways obstruction in respiratory symptomatic current and ex-smokers in primary care; 2014; San Diego, California.

[4] Perez-Padilla R, Wehrmeister FC, Celli BR, Lopez-Varela MV, Montes de Oca M, Muino A, et al (2013) Reliability of FEV1/FEV6 to diagnose airflow obstruction compared with FEV1/FVC: the PLATINO longitudinal study. PLoS One 8: e67960 10.1371/journal.pone.0067960 23936297 PMC3731337

[5] Menezes AM, Victora CG, Perez-Padilla R (2004) The Platino project: methodology of a multicenter prevalence survey of chronic obstructive pulmonary disease in major Latin American cities. BMC Med Res Methodol 4: 15 15202950 10.1186/1471-2288-4-15 PMC442126

[6] Menezes AM, Muino A, Lopez-Varela MV, Valdivia G, Lisboa C, Jardim JR, et al (2014) A population-based cohort study on chronic obstructive pulmonary disease in Latin America: methods and preliminary results. The PLATINO Study Phase II. Arch Bronconeumol 50: 10–17. 10.1016/j.arbres.2013.07.014 24332830

[7] Standardization of Spirometry, 1994 Update. American Thoracic Society. Am J Respir Crit Care Med 152: 1107–1136. 7663792 10.1164/ajrccm.152.3.7663792

[8] Hardie JA, Buist AS, Vollmer WM, Ellingsen I, Bakke PS, Morkve O (2002) Risk of over-diagnosis of COPD in asymptomatic elderly never-smokers. Eur Respir J 20: 1117–1122. 12449163 10.1183/09031936.02.00023202

[9] Perez-Padilla R, Hallal PC, Vazquez-Garcia JC, Muino A, Maquez M, Lopez MV, et al (2007) Impact of bronchodilator use on the prevalence of COPD in population-based samples. COPD 4: 113–120. 17530504 10.1080/15412550701341012

[10] Vollmer WM, Gislason T, Burney P, Enright PL, Gulsvik A, Kocabas A, et al (2009) Comparison of spirometry criteria for the diagnosis of COPD: results from the BOLD study. Eur Respir J 34: 588–597. 10.1183/09031936.00164608 19460786 PMC3334278

[11] Perez-Padilla R, Valdivia G, Muino A, Lopez MV, Marquez MN, Montes de Oca M, et al (2006) [Spirometric reference values in 5 large Latin American cities for subjects aged 40 years or over]. Arch Bronconeumol 42: 317–325. 16945261 10.1016/s1579-2129(06)60540-5

[12] Celli BR, MacNee W (2004) Standards for the diagnosis and treatment of patients with COPD: a summary of the ATS/ERS position paper. Eur Respir J 23: 932–946. 15219010 10.1183/09031936.04.00014304

[13] Pauwels RA, Buist AS, Calverley PM, Jenkins CR, Hurd SS (2001) Global strategy for the diagnosis, management, and prevention of chronic obstructive pulmonary disease. NHLBI/WHO Global Initiative for Chronic Obstructive Lung Disease (GOLD) Workshop summary. Am J Respir Crit Care Med 163: 1256–1276. 11316667 10.1164/ajrccm.163.5.2101039

[14] Menezes AMB, Perez-Padilla R, Wehrmeister FC, Lopez-Varela MV, Adriana Muiño, Gonzalo Valdivia, et al (2014) FEV1 is a better predictor of mortality than FVC: The PLATINO cohort study. PLoS One 9: e109732 10.1371/journal.pone.0109732 25285441 PMC4186841

[15] Ferguson GT, Enright PL, Buist AS, Higgins MW (2000) Office spirometry for lung health assessment in adults: A consensus statement from the National Lung Health Education Program. Chest 117: 1146–1161. 10767253 10.1378/chest.117.4.1146

[16] Watson RD, Lumb R, Young MA, Stallard TJ, Davies P, Littler WA (1987) Variation in cuff blood pressure in untreated outpatients with mild hypertension—implications for initiating antihypertensive treatment. J Hypertens 5: 207–211. 3611770 10.1097/00004872-198704000-00012

