# Supplementary material for: Instability in the COPD Diagnosis upon Repeat Testing Vary with the Definition of COPD
Source: PLoS One. 2015 Mar 26;10(3):e0121832. doi: 10.1371/journal.pone.0121832 (PMC4374954; doi:10.1371/journal.pone.0121832)
Supplement: S1 Table — (DOCX) [file pone.0121832.s002.docx]

TABLE S1- Reliability by the intraclass correlation coefficient (ICC) and 95%CI between two spirometry tests (at baseline and follow-up) in the same individuals.

| Variable | ICC (two way random effects model)* | 95%CI | ICC (one way anova)** | 95%CI |
| --- | --- | --- | --- | --- |
| PostBD FEV_1_/FVC | 0.77 | 0.74-0.79 | 0.79 | 0.78.0.80 |
| Post BD FEV_1_/FEV_6_ | 0.81 | 0.78-0.84 | 0.83 | 0.82-0.84 |
| Post BD FEV_1_/FVC<0.7 (GOLD) | 0.57 | 0.54-0.60 | 0.59 | 0.56-0.62 |
| PostBD FEV_1_/FVC<0.7 & FEV_1_%<80 (GOLD 2-4) | 0.64 | 0.61-0.66 | 0.66 | 0.64-0.69 |
| FEV_1_/FVC<LLN | 0.57 | 0.54-0.60 | 0.61 | 0.58-0.63 |
| FEV_1_/FEV_6_<LLN | 0.64 | 0.61-0.66 | 0.66 | 0.63-0.68 |
| FEV_1_/FVC<LLN & FEV_1_<LLN | 0.62 | 0.59-0.64 | 0.68 | 0.65-0.70 |
| FEV_1_/FEV_6_<LLN& FEV_1_<LLN | 0.60 | 0.57-0.62 | 0.64 | 0.62-0.67 |

Comparing 2 measurements in 2026 individuals. ICC= intraclass correlation coefficient. *= Procedure ICC from stata, two way random effects model; **Procedure loneway from STATA. PLATINO spirometry reference values.
