# Supplementary material for: Instability in the COPD Diagnosis upon Repeat Testing Vary with the Definition of COPD
Source: PLoS One. 2015 Mar 26;10(3):e0121832. doi: 10.1371/journal.pone.0121832 (PMC4374954; doi:10.1371/journal.pone.0121832)
Supplement: S2 Table — (DOCX) [file pone.0121832.s003.docx]

**TABLE S2 : Determinants of new and reversed airflow obstruction diagnosis by a logistic regression model using as criteria FEV_1_/FEV_6_<LLN**

|  | Reversed FEV_1_/FEV_6_<LLN** | | | New FEV_1_/FEV_6_<LLN** | | |
| --- | --- | --- | --- | --- | --- | --- |
|  | Odds Ratio | 95% CI | P | Odds Ratio | 95% CI | P |
| Distance of FEV_1_/FEV_6_ from the LLN (standard deviations) |  |  |  |  |  |  |
| 2-3 | 2.81 | 0.25-32 | 0.46 | 0.6 | 0.01-6.2 | 0.6 |
| 1 <2 | 5.79 | 0.6-56 | 0.13 | 2.6 | 0.3-20.9 | 0.17 |
| 0.6 <1 | 19.5 | 2-184 | 0.01 | 7.4 | 0.9-63 | 0.07 |
| <0.6 | 44.8 | 5-397 | 0.001 | 127.0 | 17-957 | <0.001 |
| Physician diagnosis of asthma | 1.08 | 0.5-2.4 | 0.80 | 1.27 | 0.7-2.4 | 0.43 |
| Smoking in the second evaluation | 0.57 | 0.25-1.3 | 0.19 | 1.48 | 0.9-2.6 | 0.17 |
| FEV_1_ post BD as score Z | 0.43 | 0.30- 0.62 | <0.001 | 1.10 | 0.8-1.5 | 0.5 |
| FET (s) first evaluation | 1.2 | 1.1-1.3 | <0.001 | 1.0 | 0.92-1.07 | 0.9 |
| FET (s) second evaluation | 0.9 | 0.8-1.0 | 0.03 | 1.1 | 1.01-1.1 | 0.01 |
| Pseudo R^2^ | 0.28 |  |  | 0.42 |  |  |

LLN= lower limit of normal, 5th percentile of the ratio according to PLATINO reference values. FET= forced expiratory time; Model was also adjusted by age and gender. PostBD =post bronchodilator. * only in the second test FEV_1_/FEV^6^ was<LLN; ** Only the first test had FEV_1_/FEV_6_<LLN. The closer the ratio and the FEV_1_ to the diagnostic threshold, the higher the risk of inconsistency on repeated testing regardless which one of the two test was abnormal. New or incident cases or resolved cases mostly are spurious and depend on the closeness to the cutpoint.
